# Symmetrical Drug-related Intertriginous and Flexural Exanthema Induced by Doxycycline

**DOI:** 10.7759/cureus.1836

**Published:** 2017-11-10

**Authors:** David G Li, Cristina Thomas, Gil S Weintraub, Arash Mostaghimi

**Affiliations:** 1 Department of Dermatology, Brigham & Women’s Hospital, Harvard Medical School

**Keywords:** sdrife, baboon syndrome, drug eruption, doxycycline, drug hypersensitivity, exanthema, pruritis

## Abstract

Symmetrical drug-related intertriginous and flexural exanthema (SDRIFE) is a cutaneous drug reaction characterized by erythema over the buttocks, thighs, groin, and flexural regions most commonly associated with the use of beta-lactam antibiotics. Although the exact pathophysiology of this disease remains unknown, it is theorized to be the result of a delayed hypersensitivity response presenting as a cutaneous eruption days to weeks after exposure to the drug. The treatment involves discontinuation of the suspected medication, symptomatic control of pruritus, and topical steroid therapy. A 51-year-old woman with homocystinuria and fibromyalgia was admitted with fevers, pancytopenia (later diagnosed to be acute myelogenous leukemia), and a targetoid cutaneous eruption in the setting of a recent tick bite. She was subsequently noted to have symmetric, pruritic, erythematous papules over the lateral neck, retroauricular regions, lateral aspects of the inframammary regions, medial upper arms, axillae, and the lower abdomen two weeks after starting doxycycline. Considering the morphology, distribution, and intense pruritis associated with the eruption, a diagnosis of SDRIFE was made. Doxycycline discontinuation along with topical steroid therapy resulted in the resolution of the eruption and pruritus. Given the widespread use of doxycycline, clinicians should be aware of this possible side effect.

## Introduction

Symmetrical drug-related intertriginous and flexural exanthema (SDRIFE) is a cutaneous drug reaction characterized by erythema over the buttocks, thighs, groin, and flexural regions. SDRIFE is most commonly associated with the use of beta-lactam antibiotics [1]. In this report, we present a case of doxycycline-induced SDRIFE.

## Case presentation

A 51-year-old woman with homocystinuria and fibromyalgia was admitted with fevers, pancytopenia, and a targetoid cutaneous eruption in the setting of a history of a tick bite. She was started on doxycycline for presumed Lyme disease. Further workup including flow cytometry and bone marrow biopsy demonstrated acute myeloid leukemia for which she was started on induction chemotherapy with daunorubicin and cytarabine.

**Figure 1:**
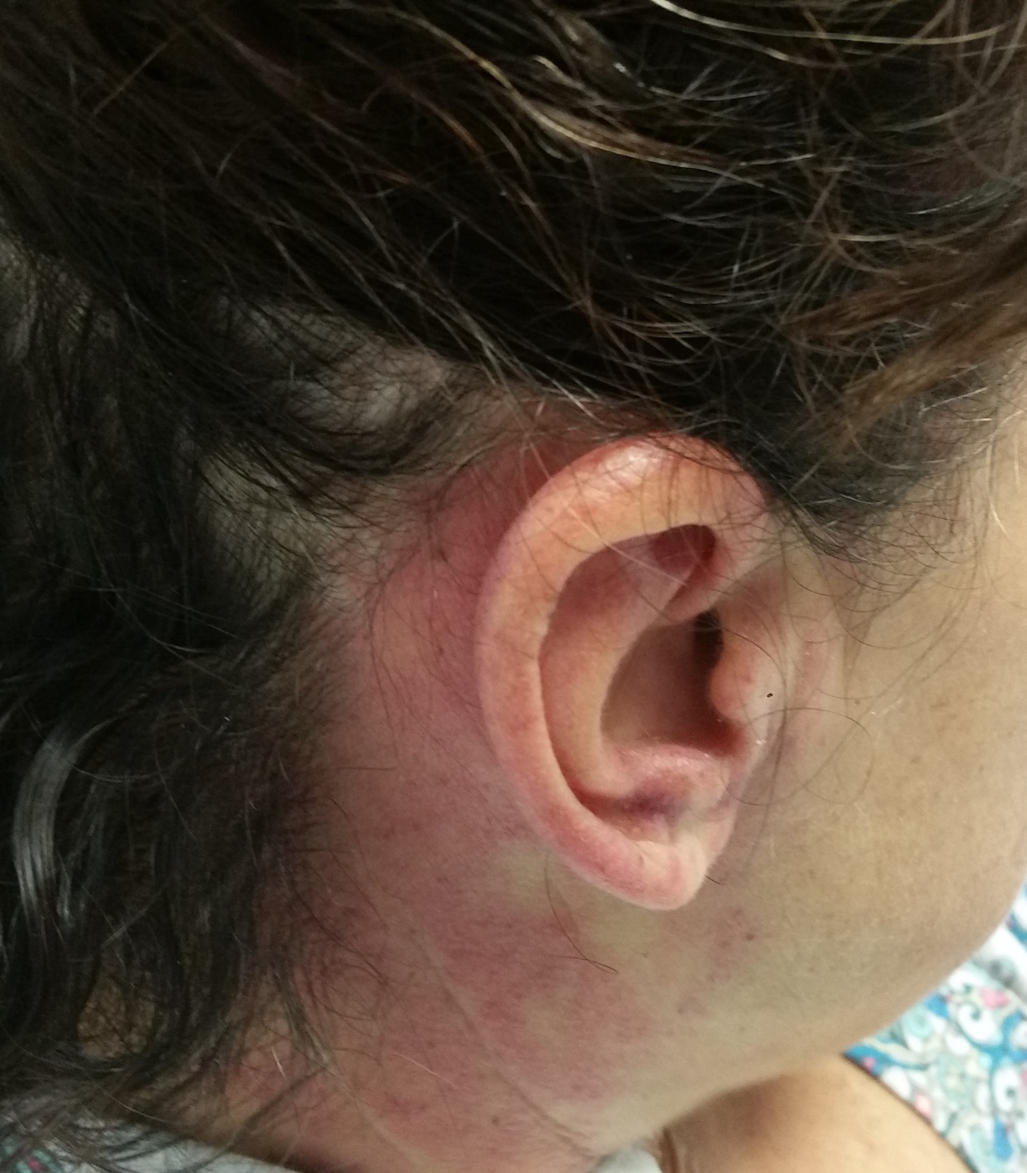
Erythematous papules and plaques occurring over the right retroauricular region

**Figure 2:**
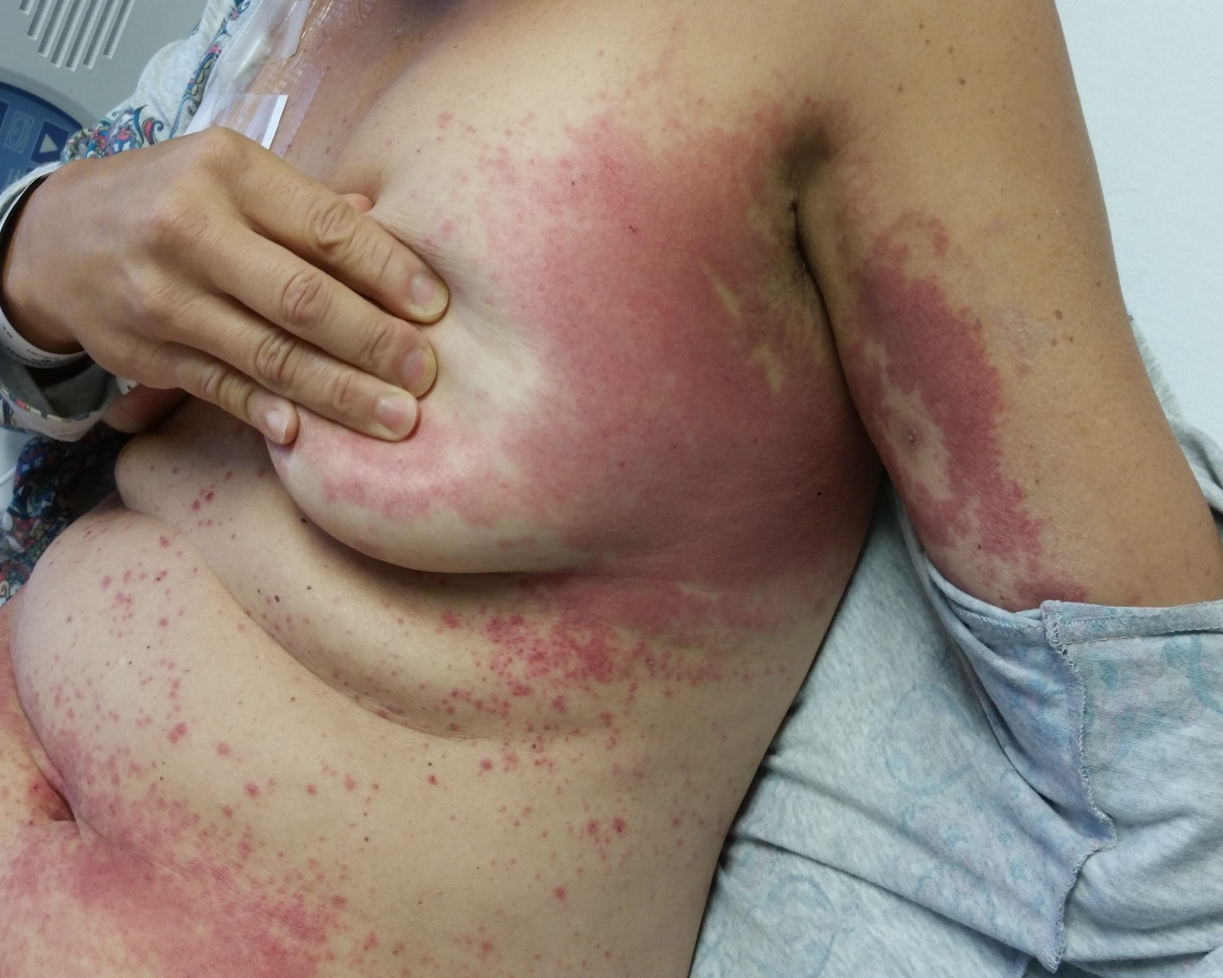
Diffusely erythematous papules and plaques with islands of sparing over the left lateral aspects of the inframammary regions, medial upper arms, and axillae

She was subsequently noted to have symmetric, pruritic, erythematous papules over the lateral neck, retroauricular regions, lateral aspects of the inframammary regions, medial upper arms, axillae, and the lower abdomen two weeks after starting doxycycline (Figures 1-2). The patient reported a similar rash during a previous treatment with doxycycline. Doxycycline was discontinued and the patient was deferred to topical steroids.

**Figure 3:**
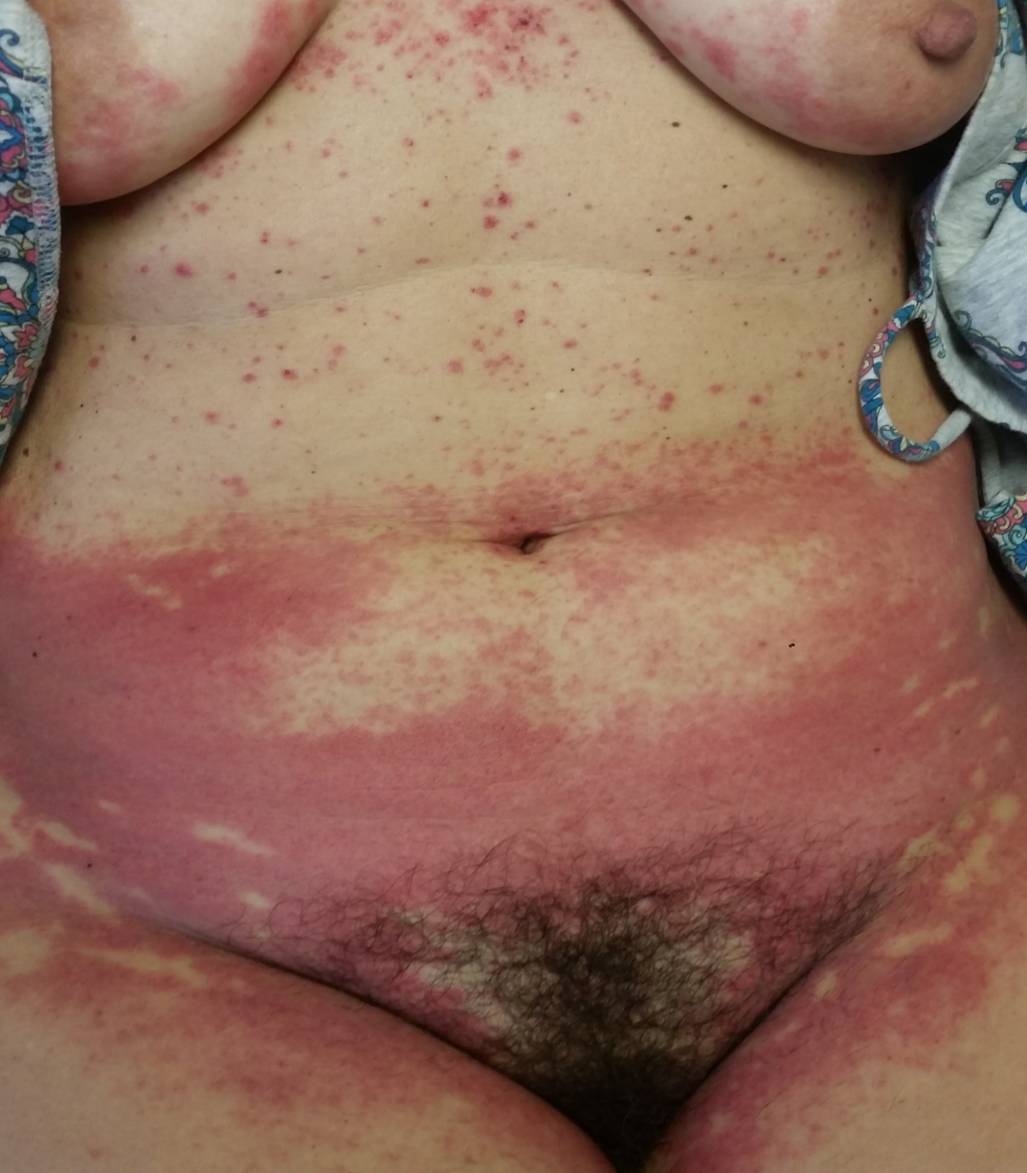
Symmetric, erythematous papules and plaques over the lower abdomen and inner thighs

The eruption had become more confluent and spread to affect the inner thighs and inner gluteal cleft five days later (Figure 3). Given the morphology and distribution of the eruption, a diagnosis of SDRIFE was made. Treatment with clobetasol 0.05% ointment and triamcinolone 0.1% ointment resulted in the resolution of the eruption and pruritus within three days.

## Discussion

Historically the term “baboon syndrome” was used to describe gluteal erythema occurring days after systemic exposure to mercury and other agents [1-2]. This terminology has been replaced by the acronym “SDRIFE”, which describes symmetric erythematous eruptions in the flexural and intertriginous folds after exposure to a systemic medication. SDRIFE is a clinical diagnosis. The proposed diagnostic criteria include exposure to a systemic medication, symmetric well-demarcated erythema of the gluteal/inguinal region and at least one other intertriginous or flexural region, and lack of systemic toxicity [1]. The most common drug association is beta-lactam antibiotics, particularly amoxicillin [1]. Other well-documented medication triggers include antihypertensives, radiocontrast media, and monoclonal antibodies [3-6]. Our patient did not have documented exposure to any of these agents.

The mechanism of SDRIFE is unknown but is hypothesized to be the result of a delayed hypersensitivity response resulting in a cutaneous eruption days after exposure to the drug [7]. Notably, skin biopsies from SDRIFE patients typically reveal a superficial perivascular infiltrate of inflammatory cells (commonly lymphocytes or eosinophils) with immunohistological studies demonstrating an infiltration of CD3+ and CD4+ T cells. These features suggest that SDRIFE shares a similar disease mechanism to type IV hypersensitivity reactions, which account for worsening of the rash after cessation of the drug [7]. The treatment involves discontinuation of the suspected agent, symptomatic control of pruritus, and topical steroid therapy.

Other relevant differential diagnoses include fixed drug eruption (FDE), acute generalized exanthematous pustulosis (AGEP), and drug rash with eosinophilia (DRESS), although the symmetric intertriginous distribution of this patient’s rash fits most closely with SDRIFE. In a patient receiving chemotherapy, it is also important to distinguish SDRIFE from toxic erythema of chemotherapy (TEC). In contrast to SDRIFE, TEC is painful and associated with desquamation. Because it represents excretion of cytotoxic agents into eccrine sweat ducts, TEC tends to be isolated to the hands, feet, and intertriginous regions [8]. In our patient, the lack of pain, absence of desquamation, and involvement of other flexural surfaces argues against TEC. Importantly, the subsequent administration of cytarabine and daunorubicin did not trigger a similar rash in this patient. We also used the Naranjo algorithm to objectively determine the probability of causality for adverse drug reactions between different agents [9]. Using this scoring method, doxycycline had a probable likelihood of causing the cutaneous reaction in our patient, whereas cytarabine and daunorubicin each had a possible likelihood.

## Conclusions

SDRIFE is a cutaneous drug reaction that is associated with beta-lactam antibiotics, anti-hypertensives, radiocontrast media, and monoclonal antibodies. To our knowledge, this is the first report of doxycycline-induced SDRIFE. Considering the widespread use of doxycycline and our report of doxycycline-induced SDRIFE, clinicians should be aware of this adverse effect.
